# Frailty prevalence and slow walking speed in persons age 65 and older: implications for primary care

**DOI:** 10.1186/1471-2296-14-86

**Published:** 2013-06-19

**Authors:** Maria-Victoria Castell, Mercedes Sánchez, Rosa Julián, Rocio Queipo, Sagrario Martín, Ángel Otero

**Affiliations:** 1 CS Dr. Castroviejo. Primary Care (SERMAS), Madrid, Spain; 2 Unidad de Medicina de Familia, Universidad Autónoma de Madrid (UAM), Madrid, Spain; 3 FIBHULP Hospital La Paz, Madrid, Spain; 4 CS Reina Victoria. Primary Care (SERMAS), Madrid, Spain; 5 Instituto de Investigación Hospital Universitario La Paz (IdiPAZ), Madrid, Spain

**Keywords:** Frailty in elderly, Walking speed, Early diagnosis, Primary care

## Abstract

**Background:**

Frailty in the elderly increases their vulnerability and leads to a greater risk of adverse events. According to various studies, the prevalence of the frailty syndrome in persons age 65 and over ranges between 3% and 37%, depending on age and sex. Walking speed in itself is considered a simple indicator of health status and of survival in older persons. Detecting frailty in primary care consultations can help improve care of the elderly, and walking speed may be an indicator that could facilitate the early diagnosis of frailty in primary care. The objective of this work was to estimate frailty-syndrome prevalence and walking speed in an urban population aged 65 years and over, and to analyze the relationship between the two indicators from the perspective of early diagnosis of frailty in the primary care setting.

**Methods:**

Population cohort of persons age 65 and over from two urban neighborhoods in northern Madrid (Spain). Cross-sectional analysis. Bivariate and multivariate analysis with binary logistic regression to study the variables associated with frailty. Different cut-off points between 0.4 and 1.4 m/s were used to study walking speed in this population. The relationship between frailty and walking speed was analyzed using likelihood ratios.

**Results:**

The study sample comprised 1,327 individuals age 65 and older with mean age 75.41 ± 7.41 years; 53.4% were women. Estimated frailty in the study population was 10.5% [95% CI: 8.9-12.3]. Frailty increased with age (OR = 1.14; 95% CI: 1.10-1.19) and was associated with poor self-rated health (OR = 2.52; 95% CI: 1.43-4.44), number of drugs prescribed (OR = 1.17; 95% CI: 1.08-1.26) and disability (OR = 6.58; 95% CI: 3.92-11.05). Walking speed less than 0.8 m/s was found in 42.6% of cases and in 56.4% of persons age 75 and over. Walking speed greater than 0.9 m/s ruled out frailty in the study sample. Persons age 75 and older with walking speed <0.8 m/s are at particularly high risk of frailty (32.1%).

**Conclusions:**

Frailty-syndrome prevalence is high in persons aged 75 and over. Detection of walking speed <0.8 m/s is a simple approach to the diagnosis of frailty in the primary care setting.

## Background

Frailty in elderly persons increases their vulnerability to stress and results in an imbalance in the body’s homeostatic reserve. Frailty weakens resistance to harmful agents, thus leading to a greater risk of disability and immobility, increased use of health services, and a higher risk of death [1-3].

Tools that facilitate early detection of vulnerable individuals at increased risk of presenting adverse effects are currently available. Different approaches to the definition of frailty, especially those related to the different frailty scales used, have made it difficult to reach a universal consensus in this regard [3-6].

However, the frailty phenotype proposed by Fried in 2001 (unintentional weight loss, exhaustion, low physical activity, slow walking speed and muscular weakness) [1], has a broad consensus and is widely used in many countries, making it possible to implement preventive and therapeutic measures to minimize the progression of frailty and its outcomes [1-3,7-9]. Its prevalence ranges from 3-6% among those aged 65–70, to over 16% among in persons aged 80 and over. Among men it has increased from 2% in those aged 65–69 to 37% in men aged 85 and over, and among women from 3% to 31%, respectively [10]. In Spain, various working groups have reported an overall prevalence of between 8.4% and 16.9% in persons aged 65 and over [11-14]. Part of this variation is due to the lack of broad consensus about the methodology used to measure this syndrome [5,13].

Several authors have stressed the role of the family physician in the detection and follow-up of older persons with frailty. This role is important both for the possibility of developing therapeutic strategies that will prevent or reverse the development of frailty, and for the opportunity to implement interventions likely to prevent adverse outcomes in frail patients, such as comprehensive geriatric assessment to optimize the treatment of comorbid conditions and promote early recognition of complications [13,15].

The widespread application in clinical practice of Fried's criteria in the primary care setting is limited by such aspects as the duration of the test, the need for measuring instruments, the cut-off points for some criteria that are not adapted to the study populations or the family physician’s heavy workload, which makes it difficult to conduct complex tests [5,15-18].

Together with frailty, slow walking speed is in itself a widely used criterion in geriatric assessment, and has become a good single estimator of frailty and its outcomes [5,19]; some authors even consider it to be a vital indicator [20]. A walking speed greater than 1.2 m/s suggests high life expectancy, whereas speeds lower than 1 m/s predict frailty and have been associated with disability, hospitalization and decreased survival [19,20]. Other authors establish the cut-off point at 0.8 m/s [21,22], while the European consensus on sarcopenia establishes a walking speed threshold of 0.8 m/s, and recommends measurement of muscle mass for anyone below this cut-off [23].

In Spain, primary care is the gateway to the public healthcare system. Care of the elderly is a priority for family physicians, given the high morbidity and frequent use of the health system in this population group. After age 75, the demand for health resources rises exponentially, along with a great increase in frailty as a syndrome [24]. Thus, the detection of frailty by the family physician can make an important contribution to the care of patients nearing the end of their lives. Given the need to optimize available resources, it is especially useful to have cost-effective clinical tools to select those individuals who can benefit most [15,16].

In this context, the present study aims to estimate frailty-syndrome prevalence and walking speed in an urban population aged 65 years and over, and to analyze the relationship between the two indicators from the perspective of early diagnosis of frailty in the primary care setting.

## Methods

### Design

Cross-sectional study based on population-based cohorts of persons age 65 and over in two urban neighborhoods of northern Madrid.

### Methodology

**Figure 1:**
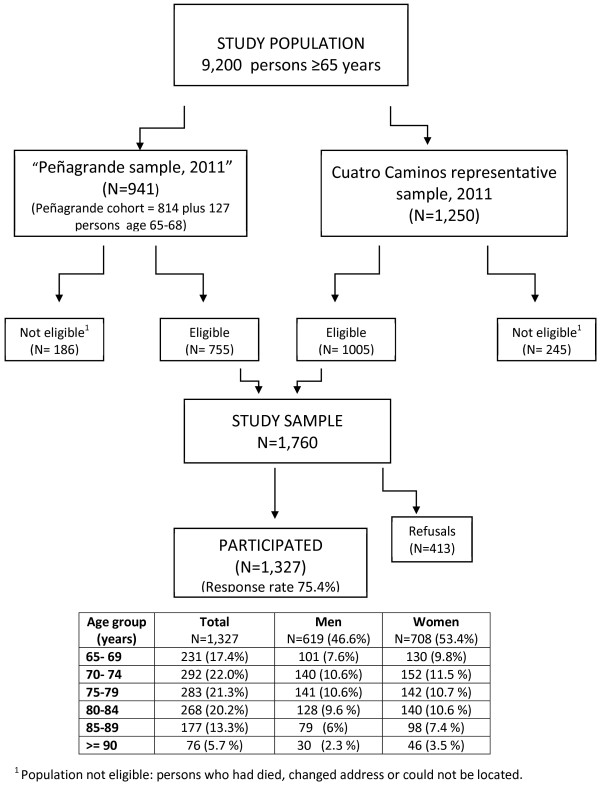
Description of the sample and level of participation.

A stratified random sample by sex and 5-year age groups was obtained of individuals aged 65 and over living in two neighborhoods in the north of Madrid (Peñagrande and Cuatro Caminos) with a combined population of 9,200 people. The data source was the population register of persons assigned to the primary health care centers in these neighborhoods [25]. The final sample consists of survivors of the Peñagrande Cohort (n = 814), created in 2008 [14], plus the random incorporation of 127 individuals age 65–68 years in 2011 (included to maintain the youngest age group), and a representative sample of 1,250 individuals from the population assigned to the Cuatro Caminos health center in 2011 (Figure 1). We obtained written informed consent for participation prior to the interview and blood sampling. The project was approved by the Clinical Research Ethics Committee in Hospital Universitario La Paz, Madrid (HULP PI 1080).

### Study variables

Frailty was evaluated according to the five criteria proposed by Fried [1], following the original cut-off points, with some adaptation: 1) *Unintentional weight loss (Shrinking)* of ≥5% in the last year: If objective information was not available, participants were asked the subjective question “Have you lost more than 3 kg in the last 3 months?” 2) *Low energy (Exhaustion):* Based on questions from the Center for Epidemiologic Studies Depression Scale (CES-D): In the last week “I felt that everything I did was an effort" and "I couldn´t get going. Those who answered "frequently" or "always" to at least one of these questions were considered to meet this criterion. 3) *Weakness*: Grip strength in the dominant hand was measured with a dynamometer (Jamar TM Hidraulic Hand Dynamometer, Preston, Jackson, Missouri, EEUU) adjusted for body mass index (BMI) according to the lowest quintile. The cut-off points established were, for men: BMI ≤24 and grip strength <18.5 kg; 24 < BMI ≤28 and grip strength <20 kg; BMI >28 and grip strength <22 kg; and for women: BMI ≤29 and grip strength <11 Kg; BMI >29 and grip strength <12 kg. 4) *Slowness:* Calculated after walking 3 meters, adjusted for sex and height, according to Fried. The cut-off points for 3 meters were established as, in men: height ≤173 cm and time ≥4.59 s (equivalent to 0.65 m/s); height >1.73 cm and time ≥3.93 s (equivalent to 0.76 m/s); and for women: height ≤1.59 cm and time ≥4.59 s (0.65 m/s); and height >1.59 cm and time ≥3.93 s (0.76 m/s). 5) *Low physical activity*: Kilocalories (kcal) expended per week were calculated based on the Longitudinal Ageing Study Amsterdam (LASA) Physical Activity Questionnaire (LAPAQ) [26], which is used to record daily physical activity (walking, cycling, light and heavy household chores and gardening) and physical exercise. We maintained the cutoffs proposed by Fried: <383 kcal/week for men and <270 kcal/week for women. Persons who met at least three criteria were considered to be frail.

Walking speed, considered as a separate indicator, was expressed in m/s and was obtained at the same time as the slowness criterion was measured.

Independent variables. The sociodemographic variables measured were age, sex and marital status, number of cohabitants (alone or accompanied), educational level (low if less than primary education; medium if primary but not secondary education is completed; high if secondary or university level education), social status (low status: agricultural worker, unskilled manual worker or housewife with low educational level; medium status: skilled manual worker, self-employed worker or housewife with medium or high educational level; high status: managers, professionals), and socioeconomic level (total household monthly income with two categories and cut-off point at 600 euros (about $ 760). Health status was measured by the following variables: Self-rated health, which was defined based on the question “How is your health in general?” (good if the answer was fair, good or very good, and poor if the answer was poor or very poor); Obesity (body mass index or BMI = weight in kilograms/height in meters squared equal to or higher than 30); Comorbidity, if there was presence of two or more chronic diseases from a list of seven which includes: respiratory problems, heart disease, peripheral artery disease, diabetes mellitus, stroke, cancer and osteoporosis [27]; Cognitive decline, when the person obtained a score of ≤24 on the Mini Mental State Examination (MMSE) validated for the Spanish population [28]; Disability was considered to exist if the person could not carry out at least one of the following basic activities of daily living without help: walking across a small room, bathing or showering, personal grooming and getting dressed. Finally, medication used in the last 2 weeks for both acute and chronic diseases was recorded. “Polypharmacy” was considered to be current intake of 5 or more medications [29].

### Statistical analysis

Frequencies and their 95% confidence intervals (95% CI) were calculated for qualitative variables. The mean, standard deviation and range were obtained for quantitative variables. For the sole purpose of estimating data in the reference population, the data were weighted by assigning a specific weight (W) to each individual in the sample, calculated as W = Nexp/Nobs, where Nobs is the number of persons in a specific age/sex category in the cohort, and Nexp is the number of persons of a specific sex and age in the north district of Madrid in 2010. The prevalence of the frailty syndrome and walking speed <0.8 m/s were calculated.

A bivariate analysis of frailty was made of the sociodemographic and analytic variables, using the chi-square test for categorical variables and Student’s t-test for continuous variables. Statistical significance was considered to be a p-value <0.05. A multivariate analysis with binary logistic regression was performed using those variables that were associated with frailty in the bivariate analysis with a p-value <0.10. A backward stepwise procedure (back-step) was used to eliminate variables in the model. Age and number of drugs were introduced in the model as continuous variables after verifying that they met the criterion of linearity. Each factor was tested for interaction with age and sex. The quality of the adjustment of the final model was assessed using the Hosmer-Lemeshow goodness-of-fit test and Nagelkerke’s coefficient of determination.

To study the characteristics and distribution of the indicator walking speed in the study population, expressed in meters/second to walk 3 meters, we selected different cut-off points to classify each person as slow or not slow. To cover the range of the different proposals in previous publications [13,16] we used the following cut-off points: 0.4, 0.5, 0.6, 0.7, 0.8, 0.9 ,1.0, 1.1, 1.2, 1.3 and 1.4 m/s. To analyze the relationship between the two indicators, frailty and walking speed, we calculated the likelihood ratios (LR) [30]. The LR + and LR- permit calculation of the probability of frailty in patients with and without slow walking speed (SWS), respectively. (LR + =% of people with SWS who are frail /% of people with SWS who are not frail; LR- =% people without SWS who are frail /% people without SWS who are not frail).

The statistical analysis was performed using the software package SPSS 19.0 for Windows.

## Results

The study sample was made of up 1,327 individuals aged 65 years and over from the Madrid neighborhoods of Peñagrande and Cuatro Caminos (Figure 1). The mean age was 75.4 ± 7.4 (range: 65–104), and 53.4% were women (708/1327).

Table 1 shows the distribution of the variables in the study population. The frailty syndrome was present in 148 of 1,325 participants (frailty could not be measured in two persons). The estimated population prevalence of frailty was 10.5% [95% CI: 8.9-12.3] (data weighted by age and sex). The prevalence was higher in women (13.7%; 95% CI: 11.4-16.3) than in men (6.0%; 95% CI: 4.2- 8.4) and increased with age. The mean age of non-frail individuals was 74.8 ± 6.8 while that of frail persons was 85.9 ± 7.8.

**Table 1:** Distribution of variables in the study population by frailty (data weighted by age and sex)

	Total^*^	Not frail**	Frail**	P value***
	**% (95% CI)**	**% (95% CI)**	**% (95% CI)**	
	**N = 1325**	**N = 1177**	**N = 148**	
**Age**				**N = 1325**
<75 (N = 523)	48.5 (45.8- 51.3)	98.6 (97.3- 99.3)	1.4 (0.7- 2.7)	
≥75 (N = 804)	51.5 (48.7-54.2)	80.9 (77.7- 83.7)	19.1 (16.2- 22.3)	
**Sex**				<0.001
Women (N = 708)	58.7 (55.9- 61.2)	86.3 (83.6- 88.6)	13.7 (11.4-16.3)	
Men (N = 619)	41.3 (38.8- 44.1)	94.0 (91.6- 95.8)	6.0 (4.2-8.4)	
**Educational level**				<0.001
Complete primary (N = 835)	66.5 (63.8- 69.1)	92.9 (90.9- 94.5)	7.1 (5.5-9.0)	
Incomplete primary (N = 449)	33.5 (30.9- 36.2)	85.1 (81.3- 88.2)	14.9 (11.7-18.7)	
**Marital status**				<0.001
Married/with partner (N = 807)	60.3 (57.5- 62.8)	93.5 (91.5- 95.0)	6.5 (4.9-8.5)	
Single/Separated (N = 163)	13.6 (11.7-15.5)	89.9 (84.3- 93.8)	10.1 (6.2-15.7)	
Widowed (N = 352)	26.1 (23.9-28.8)	80.2 (75.5- 84.2)	19.8 (15.7-24.4)	
**Socioeconomic status**				<0.001
Hight-medium (N = 768)	59.0 (56.2- 61.6)	92.2 (90.1-93.9)	7.8 (6.0-9.9)	
Low (N = 547)	41.0 (38.4- 43.8)	85.9 (82.6- 88.6)	14.1 (11.3-17.5)	
**Lives alone**				0.133
No (N = 1057)	79.8 (77.85- 81.9)	89.0 (86.9-90.7)	11.0 (9.2-13.1)	
Yes (N = 263)	20.2 (18.2-22.6)	92.1 (88.0-94.9)	7.9 (5.1-11.9)	
**Self-rated health**				<0.001
Good (N = 1154)	89.7 (87.9- 91.3)	92.5 (90.8- 93.9)	7.5 (6.0-9.2)	
Poor (N = 133)	10.3 (8.7-12.1)	69.7 (61.0- 77.2)	30.3 (22.8-39.0)	
**Comorbidity**				<0.01
<2 diseases (N = 866)	66.2 (63.6- 68.7)	91.4 (89.33- 93.2)	8.6 (6.8-10.7)	
≥ 2 diseases (N = 461)	33.8 (31.3-36.4)	85.9 (82.2- 88.9)	14.1 (11.1-17.8)	
**BADL**				<0.001
Abled (N = 1170)	89.5 (87.6- 91.0)	95.5 (94.1- 96.6)	4.5 (3.42-5.9)	
Disabled (N = 151)	10.5 (9.0-12.4)	38.1 (30.1- 46.8)	61.9 (53.2-69.9)	
**Use of medication**				<0.001
<5 medications (N = 561)	44.3 (41.6- 47.0)	95.1 (92.9- 96.6)	4.9 (3.4-7.1)	
≥ 5 medications (N = 766)	55.7 (53.0-58.4)	85.1 (82.2- 87.5)	14.9 (12.5-17.7)	
**Cognitive decline**				<0.001
No (N = 1106)	84.4 (82.3- 86.3)	93.2 (91.5- 94.6)	6.8 (5.4-8.5)	
Yes (N = 217)	15.6 (13.7-17.7)	70.9 (64.1- 76.9)	29.1 (23.2-36.1)	
**Obesity**				<0.05
BMI ≤25 (N = 260)	20.4 (18.3- 22.8)	88.8 (84.1- 92.2)	11.2 (6.7-14.5)	
BMI > 25 BMI < 30 (N = 594)	46.2 (43.4- 48.9)	93.7 (91.3- 95.4)	6.3 (4.6-8.7)	
BMI ≥30 (N = 405)	33.4 (30.8-36.1)	93.1 (90.1- 95.3)	6.9 (4.7-9.8)	
**TOTAL**		89.4 (87.5- 90.9)	10.5 (8.9- 12.3)	

*% of each category within variable; **% frail/not frail; ^***^ P-value: χ2 Test.

*BADL* Basic Activities of Daily Living, *BMI* Body Mass Index.

The prevalence of frailty was 19.1% [95% CI: 16.2-22.3] in persons age 75 and older and 1.4% [95% CI: 0.7-2.7] in those under 75 years. Frailty was more frequent in women, those aged 75 and over, widows, persons with low educational level and socioeconomic status, poor self-rated health, comorbidity, polymedication, disability and cognitive decline (Table 1).

The multivariate analysis (Table 2) shows the association between frailty and age (mean increase in odds of frailty of 14% for each additional year of life). Frailty was also associated with poor self-rated health (OR 2.52; 95% CI: 1.43-4.44), number of drugs prescribed (17% increase in odds of frailty for each additional drug), and disability (OR 6.58; 95% CI: 3.92-11.05) after adjusting for all the variables that showed an association in the bivariate analysis.

**Table 2:** Multivariate analysis of frailty

	**Model I**	**Model II**
	**OR [95% CI]**	**OR [95% CI]**
**Age **^a^	1.21 [1.18- 1.25]	1.14 [1.10- 1.19]
**Sex**: Woman (reference: man)	1.94 [1.23- 3.05]	1.30 [0.82- 2.06]
**Poor self-rated health** (reference: good)		2.52 [1.43- 4.44]
**Number of drugs **^a^		1.17 [1.08- 1.26]
**Disability** (reference: capable of BADL)		6.58 [3.92- 11.05]

^a^ Quantitative variable. *BADL* Basic activities of daily living.

Analysis adjusted for:

Model I (age, sex, educational level, socioeconomic level). R^2^ =0.36.

Model II (age, sex, self-rated health, comorbidity, number of drugs, cognitive decline, physical disability). R^2^ = 0.45.

The variables not shown in the table were not significantly associated with frailty.

Table 3 presents the distribution of walking speed in the cohort by different cut-off points and its association with frailty. A walking speed of less than 0.8 m/s was presented by 42.6% of cases in the total sample, 99.3% of the frail and 35.5% of the non-frail (LR + = 2.80 overall). In the subpopulation of 75 and over the proportion of people with walking speed <0.8 m/s was 56.4% (452/802) with a LR + =2.10. A walking speed of less than 0.9 was found in 50.2% (100% of frail and 43.9% of non-frail individuals) (LR + overall = 2.28 overall). In the subpopulation of 75 and over the proportion of people with walking speed <0.9 m/s was 63.7% (511/802) with a LR + =1.78.

**Table 3:** Distribution of walking speed by different cut-off points in the cohort

**Walking speed (m/s)**	**Total**	**Frail**	**Non frail**	**LR +**	LR + ≥75*	**LR -**	LR- ≥75*
	**N = 1325**	**N = 148**	**N = 1177**				
<0.4	136 (10.3%)	86 (58.1%)	50 (4.2%)	13.83	8.66	0.44	0.44
<0.5	185 (14.0%)	101 (68.2%)	84 (7.1%)	9.60	6.68	0.34	0.34
<0.6	278 (21.0%)	120 (81.1%)	158 (13.4%)	6.04	4.24	0.22	0.24
<0.7	400 (30.2%)	138 (93.2%)	262 (22.3%)	4.19	3.00	0.09	0.10
<0.8	565 (42.6%)	147 (99.3%)	418 (35.5%)	2.80	2.10	0.01	0.01
<0.9	665 (50.2%)	148 (100.0%)	517 (43.9%)	2.28	1.78	0.00	0.00
<1.0	766 (57.8%)	148 (100.0%)	618 (52.5%)	1.90	1.56	0.00	0.00
<1.1	1001 (75.5%)	148 (100.0%)	853 (72.5%)	1.38	1.20	0.00	0.00
<1.2	1062 (80.2%)	148 (100.0%)	914 (77.7%)	1.29	1.15	0.00	0.00
<1.3	1132 (85.4%)	148 (100.0%)	984 (83.6%)	1.20	1.10	0.00	0.00
<1.4	1178 (88.8%)	148 (100.0%)	1028 (87.3%)	1.14	1.07	0.00	0.00

*LR+*, Likelihood Ratio Positive; *LR-* Likelihood Ratio negative.

*LR in individuals aged 75 and over.

Frailty could be ruled out in 99.9% of individuals in our sample aged 75 and over with walking speed ≥0.8 m/s (LR- = 0.01), and in 100% of those with walking speed ≥0.9 m/s. Other cut-off points for walking speed show similar differences in prevalence and LR (+ or -) in its relation with frailty, with a higher probability of an association with frailty at lower walking speeds and a lower probability with higher walking speeds (Table 3).

## Discussion

Frailty as a geriatric syndrome entails an increased risk of falls, functional decline, hospitalization and death, as well as greater use of health and social resources [5,6,31]. Fried’s phenotype, based on the measurement of five criteria, enjoys broad consensus in the scientific community. General practitioners who need a simpler approach to frailty evaluation may find assessment of the frailty phenotype to be more feasible [15].

The prevalence of frailty in our population according to Fried’s criteria is around 10.5% in persons age 65 and older, which is consistent with studies in other Spanish populations [11-13] as well as large studies in other countries [7,8]. In our study frailty is more frequent in women (13.7% versus 6.0%) and occurs at an earlier age than in men. Women's poorer level of health is largely responsible for this: they have greater cognitive decline, more disability and greater use of drugs, which translates into higher comorbidity. Women report worse self-rated health than men, and this parameter is also associated with frailty [9]. Socioeconomic and health differences between men and women are becoming narrower with new generations of older people [29]. However, some sociodemographic and health characteristics still require a clinical approach differentiated by sex.

The association of frailty with age has been clearly established [1,8,11,13,32]. Our study shows an average 14% increase in the odds of frailty in the population for each year of life after age 65, resulting in a prevalence of 19% in those aged 75 and over. The increase is more pronounced after age 85, 40.2% of whom are frail. This is consistent with the delayed onset of adverse health events in the geriatric population that has been observed based on multiple health parameters [24]. In any event, consideration of frailty is important to better reflect biological age [5].

Despite the overlap between frailty, disability and comorbidity, they represent distinct domains that are not interchangeable [2,14]. As shown in our study, they are often associated, but the detection of frailty has an important impact on care in the last stage of life, precisely because it does not focus on the diagnosis of illnesses [15].

Early diagnosis of frailty in primary care is an important goal – first, because of its high prevalence, which is likely to rise even further in the future [16], second, because of its prognostic value, given these individuals' greater risk of presenting functional impairment or adverse health events [11-16], and finally, because there are possible treatments that can delay or even reverse frailty in its early stages, before the onset of physical and/or mental disability [15,24]. When clinicians are considering any changes in patient care, including a new medication, they should not only evaluate the patient’s morbidity, but should also take into account the individual's degree of frailty and the extent to which the intervention may affect the prognosis [3].

However, diagnosis of the frailty phenotype in daily practice has some drawbacks since the original cut-off points for some criteria cannot always be extrapolated to different populations [11,13,32]; moreover, it is based on a combination of five tests, which require instruments and take over 10 minutes to perform [16]. Thus, a test that is easy to perform and is highly related to frailty may be of interest. Walking speed is a simple, quick, and easily performed test [19]. It is widely used in comprehensive geriatric assessment [23] and, unlike Fried's slowness criterion, does not require adjustment for either sex or height. Some investigators have found that walking speed alone is a health indicator that is particularly informative after age 75 years [20] and is predictive of frailty and adverse outcomes [5,7].

In our study, frailty in individuals aged 65–74 years is very low (around 1.4%) and increases among those aged 75 and over to 19%. Based on these data, exploration of the five items that make up the frailty syndrome in all persons aged 75 and over would result in a diagnosis of frailty in one of every five individuals examined. Such an effort would involve a heavy burden of work given the large number of persons targeted.

The cut-off point to identify persons with slow walking speed is a subject of debate; the most frequently employed cut-offs are 1 m/s and 0.8 m/s [20-23]. In our analysis of multiple cut-off points (between 0.4 and 1.4 m/s) we found that between 0.8 and 0.9 m/s is the most useful cut-off above which frailty can be ruled out. The prevalence results found in those cut-off points, as well as the LR + and LR-, which are shown in Table 3, and the fact that 0.8 m/s has been proposed by different authors [22,23] inclines us to choose that cut-off point as the one most closely related to the diagnosis of frailty.

**Figure 2:**
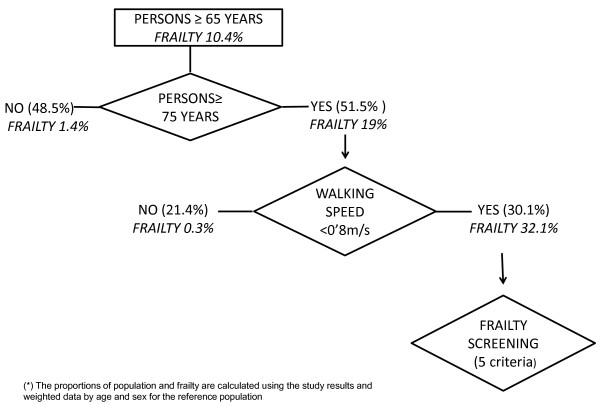
Proposed algorithm for diagnosis of frailty in primary care based on age and walking speed.

In this context, and based on our results, we propose a simple approach to the diagnostic process in the primary care setting. Figure 2 shows an algorithm for action based on the distribution of the elderly population, the estimation of the prevalence of frailty in the different study groups, and the capacity of slow walking speed to predict the presence of frailty.

A critical aspect with respect to implementation of this measure in primary care is the applicability of the test; its reproducibility must be assured. Typically, it is measured over a distance of 5 m, although it may need to be adapted in primary care consultations to 3 or 4 meters, depending on space availability [5,33].

With regard to the study limitations, aside from those characteristic of any cross-sectional study, it should be taken into account that many of the variables used were self-reported, although frailty was measured based on an objective test performed by health personnel. The fact that the study was conducted in a large population-based cohort (1,327 participants) representative of the older population in an urban area supports the strength of the results. It should be noted that our work was not meant to be a validation study, since the two indicators compared were measured at the same time and in the same population. The LR was chosen to overcome this weakness in the field work design. As noted by Fletcher and Fletcher, the likelihood ratio, in this context, contains information similar to sensitivity and specificity without the design of a validation study [30].

## Conclusions

Persons aged 75 years and over have a high prevalence of frailty, which reached 19% in our study. Early diagnosis of frailty by the family physician has clear implications for improving health given the possibility of reversing the process and preventing adverse events in the elderly. However, exploration of Fried’s five criteria requires use of a substantial amount of resources in primary care.

Measurement of walking speed is a simple, quick, and easily performed test that is a good indicator of health and survival in older adults, especially after age 75. Our data confirm that a walking speed of ≥0.9 m/s rules out the presence of frailty, and that a walking speed of ≤0.8 m/s doubles the probability of a diagnosis of frailty. Accordingly, we propose that the first step in frailty detection should be measurement of walking speed in all persons aged 75 and over in the family physician’s daily clinical practice, using a cut-off point of 0.8 m/s. A standardized methodology must be used to ensure reproducibility.

### Ethical approval

All patient registration data were treated confidentially according to the Spanish Organic Law on Data Protection of 1999. No electronic patient identifier was used.

## Competing interests

The authors declare that they have no competing interests.

## Authors’ contribution

Conceived and designed the field work: MVC, MS, AO. Performed the field work: MVC, RJ, SM. Analyzed the data: MVC, RQ, AO. Contributed reagents/materials/analysis tools: RQ. Wrote the paper: MVC, AO, MS. All the authors contributed ideas, revised different manuscript versions and approved the final one.

## Pre-publication history

The pre-publication history for this paper can be accessed here:

http://www.biomedcentral.com/1471-2296/14/86/prepub
